# Non-local mean denoising in diffusion tensor space

**DOI:** 10.3892/etm.2014.1764

**Published:** 2014-06-06

**Authors:** BAIHAI SU, QIANG LIU, JIE CHEN, XI WU

**Affiliations:** 1 Department of Nephrology, West China Hospital, Sichuan University, Chengdu, Sichuan 610041, P.R. China; 2 Department of Computer Science, Chengdu University of Information Technology, Chengdu, Sichuan 610225, P.R. China

**Keywords:** diffusion tensor imaging, denoising, non-local mean

## Abstract

The aim of the present study was to present a novel non-local mean (NLM) method to denoise diffusion tensor imaging (DTI) data in the tensor space. Compared with the original NLM method, which uses intensity similarity to weigh the voxel, the proposed method weighs the voxel using tensor similarity measures in the diffusion tensor space. Euclidean distance with rotational invariance, and Riemannian distance and Log-Euclidean distance with affine invariance were implemented to compare the geometric and orientation features of the diffusion tensor comprehensively. The accuracy and efficacy of the proposed novel NLM method using these three similarity measures in DTI space, along with unbiased novel NLM in diffusion-weighted image space, were compared quantitatively and qualitatively in the present study.

## Introduction

Diffusion tensor imaging (DTI) has evolved into a primary technique for characterizing the structure and architecture of living tissue non-invasively *in vivo* (1). The Brownian motion of free water molecules is first measured using diffusion sensitizing gradients along different spatial directions to generate diffusion-weighted images (DWIs), from which DTI data are reconstructed under the assumption of a Gaussian model. Mathematically, the tensor is represented by a 3×3, symmetric positive definite matrix, whose eigenvalues and eigenvectors describe the magnitudes and directions of water diffusion in each voxel imaged. The eigenvector corresponding to the largest eigenvalue is assumed to be along the longitudinal axis of white matter fiber bundles, the integration of which over the image volume allows the trajectories of the white matter fiber pathways to be delineated *in vivo* (2). An additional important parameter, fractional anisotropy (FA), which is derived from the eigenvalues, has been found to be a very sensitive index of the structural integrity of brain tissue (3).

The unique capability of DTI has been exploited in the past decade in a number of clinical studies that aimed to understand the structural basis of functional anomalies or the normal developmental processes of the human brain (4,5). Despite the success in providing insight into the tissue microstructure and architecture, the reliability and validity of DTI, particularly in the context of tractography, remain a common concern. Most notably, due to the use of echo planar imaging sequences, the DWIs from which tensors are reconstructed are particularly vulnerable to imaging noise, which necessarily imparts the parameters subsequently derived from the DTI data (3).

To improve the reliability and validity of DTI applications, extensive efforts have been made to suppress image noise by image smoothing. To date, the smoothing methods reported are primarily neighborhood based and may be broadly classified into two categories: Denoising in the DWI space (6–10) and denoising in the DTI space (11,12). Although these methods have been demonstrated to be able to reduce the noise effectively, each method has advantages and disadvantages. Denoising in the DWI space enhances the signal-to-noise ratio prior to tensor reconstructions, but these methods provide no guarantee to the smoothness of the DTI space since higher level constraints from the structure information cannot be elegantly embedded. In addition, denoising in DWI space requires restoring the images for all spatial directions. This duplication markedly increases the computation time, particularly in high angular resolution diffusion Imaging (HARDI), which involves hundreds of directions for restoration (13). By contrast, denoising in the DTI space usually smooths the tensor space using tensor elements or the decomposed tensor spectra. While methods in this category possess the advantage of being able to maintain explicitly the coherence of structures inherent in the image, they are dominantly based on limited features derived from the tensor and, thus, impose constraints in only a sub-space of the DTI.

In the present study, a novel DTI space denoising method, based on recently established non-local mean (NLM) smoothing (14), was proposed. In contrast to the conventional NLM denoising method, which smooths images in a gray-level scale, this method adapted NLM into DTI space. In addition, the weighting scheme of NLM was implemented using affine invariant metrics, which compare geometric and orientation features of the diffusion tensors comprehensively. The benefits of the proposed method are three fold. Firstly, using NLM denoising in DTI space ensures an optimal restoration result for the geometric and orientation information of the diffusion tensor. Secondly, comparison with affine invariant metrics produces a more accurate weighting scheme and, thus, involves further improvement of NLM denoising. Finally, computational redundancy is markedly decreased for tensor base smoothing and the effective weighting scheme.

The present study introduces the basic principle of this smoothing method, as well as the implementation details. Performance of the algorithm was evaluated on the basis of three similarity measures, namely, Log-Euclidean distance (LED) (15), Riemannian distance (RD) (16) and Euclidean distance (ED) (17), together with the unbiased NLM method in DWI space (10). Experimental results with synthetic and real *in vivo* DTI data were assessed.

## Materials and methods

### Methods

The original NLM algorithm proposed by Buades *et al* (14) smooths gray-level images on the basis of image intensity similarity, instead of spatial proximity, which is formulated under the framework of the Markov random field (18). Specifically, for position *p*, the filtered intensity *I* is computed as follows (equation 1);

$$I(p)=\sum_{q\in \Omega }w(p,q)v(q)$$

where *p* and *q* are the positions of image pixels, *v* is the noised image and *Ω* is a neighborhood of *p* with a reasonable size. The parameter *w* is a weighting factor computed as follows (equation 2):

$$w(p,q)=\frac{1}{Z(p)}\text{exp}(-\frac{d^{2}(p,q)}{h^{2}})$$

Where *d(p,q)* is an ED of the gray level between pixels *p* and *q*, *h* controls the rate of decay of the exponential function and *Z* is a normalizing factor calculated as follows (equation 3):

$$Z(p)=\sum_{q\in \Omega }\text{exp}(-\frac{d^{2}(p,q)}{h^{2}})$$

According to the aforementioned equations, the estimated value of *I* is a weighted average of the pixels in the *Ω* neighborhood, where the pixels with more similar gray levels are assigned larger weights. Essentially, the NLM reduces noise by exploiting the self-similarity of image gray levels, which has been demonstrated to be an effective method of imaging smoothing for conventional MRI (19), as well as DWIs (12).

Unlike MRI or DWI generation, DTI uses a symmetric positive definite matrix in each voxel to describe the tensor model, which characterizes unique microstructural geometric information in white matter fiber bundles. Therefore, DTI comparison is required to characterize variability based on the entire tensor and thus, is defined as the distance between them. To define the geometric distance between tensors, metric and local coordinate systems for tensor representation are required. Basser and Pajevic (20) proposed a tensor-variate statistical framework which placed the diffusion tensor on a Euclidean manifold (equation 4):

where ||•|| denotes the Frobenius norm. This Euclidean metric is defined over an entire space of symmetric matrices and is rotation invariant, which makes it invariant for the selection of orthogonal coordinates.

$$d_{Euc}(V(p),V(q))=\|V(p)-V(q)\|$$

An additional framework is Riemannian metric, which is affine invariant and operates only on tensors belonging to the space of positive definite symmetric matrices (equation 5) (16):

$$d_{Riemann}(V(p),V(q))=\|\text{log}(V{\left(p\right)}^{-1}V(q))\|=\sqrt{\sum_{i=1}^{3}{\text{ln}}^{2}\;\lambda_{l}}$$

where *V* is the tensor matrix and *λ_i_* is the i^th^ eigenvalue of the matrix, *V*(*p*)^−1^*V*(*q*). Compared with the Euclidean metric, which admits non-positive tensors and exhibits a swelling effect, the affine invariant metric coincides with the Fisher information metric (21) and Kullback-Leibler divergence (22), and is proposed as a natural metric for DTI. In addition, the Log-Euclidean metric, with its corresponding geodesic, was proposed as an efficient approximation for the computationally demanding affine-invariant metric and is implemented in a number of tensor processing applications (equation 6) (15):

$$d_{LogEuclidean}(V(p),V(q))=\sqrt{Trace[{\left(\text{log}(V(p))-\text{log}(V(q))\right)}^{2}]}$$

where *Trace* denotes the sum of the eigenvalues.

According to the original NLM method (14), the gray-level intensities of an image are assumed to be approximate with normal distribution. However, since the diffusion tensor space is not Euclidean, the normal distribution can be adopted into log-normal distribution and the first equation should be rewritten as the geometric mean (equation 6):

$$V(p)=\text{exp}(\sum_{q\in Q}w(p,q)\;\text{log}(V(q)))$$

where the weight can be calculated using equations 4–6

### Experiments with simulated DTI data

**Figure 1:**
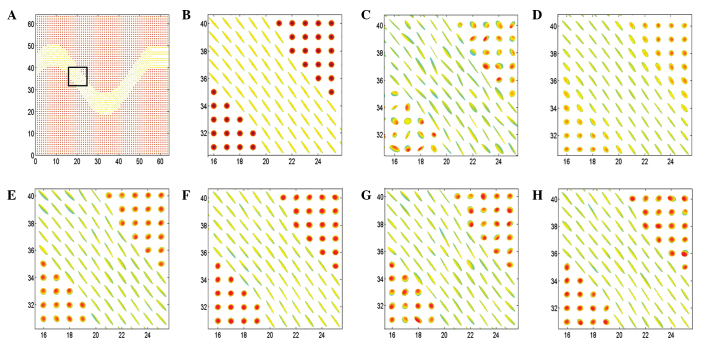
Visual assessment of the denoising effects on synthetic DTI data. (A) Synthetic dataset without noise. (B) Enlarged view of the boxed region in (A). Same region of (B) with (C) 5% Rician noise, and denoising with (D) GF, (E) RD, (F) LED, (G) ED and (H) UNLM in DWI space. DTI, diffusion tensor imaging; GF, Gaussian filtering; RD, Riemannian distance; LED, Log-Euclidean distance; ED, Euclidean distance; UNLM, unbiased non-local mean; DWI, diffusion weighted image.

The proposed algorithm was first evaluated with a synthetic DTI dataset, which was designed to have a sinusoid geometric structure, as shown in Fig. 1A. To improve the visualization details of the synthetic fibers, the boxed region in Fig. 1A is shown in an enlarged view in Fig. 1B. Each voxel was visualized by an ellipsoid, whose principal axes were the three orthogonal eigenvectors of the tensor, and the radii of the ellipsoid along the axes were proportional to their corresponding eigenvalues. To closely mimic the physiological *in vivo* conditions, synthetic tensors in the curves were constructed to have a trace of 2.1×10^−5^ cm^2^/sec and an FA of 0.8. These diffusion parameters were similar to those in normal brain parenchyma. Diffusion-weighted imaging was simulated along 32 non-collinear directions with a *b* value of 1,000 sec/mm^2^, and the diffusion-weighted data were corrupted with 5% Rician noise as follows (equation 8):

$$s_{i}=\sqrt{{\left(a_{i}+x_{i}\right)}^{2}+{y_{i}}^{2}}$$

where *a_i_* is the simulated DWI signal at pixel *i,* and *x_i_* and *y_i_* are both Gaussian noise with a mean value of zero and identical standard deviations of 0.05. Fig. 1C demonstrates an example of noised DTI tensors whose principle direction and shape are disarranged due to noise corruptions.

The proposed DTI-NLM method using ED (equation 4), RD (equation 5) and LED (equation 6) was examined with a synthetic DTI dataset. In addition, an unbiased non-local mean (UNLM) algorithm in DWI space was used (10) and calculated to tensor using a linear least-square fitting procedure for comparison (1).

Three approaches were employed to compare the performance of these denoising methods, namely, visual assessment, quantification of the mean angular deviation of the principal direction (PD) and mean deviation of the FA prior to and following denoising. The first assessment demonstrated the improvement of the visual effect with the denoising methods. The second assessment measured the capability of these denoising methods to restore the tensor orientation, while the last assessment gauged the extent to which the tensor directionality was restored with the denoising methods.

Finally, the effects of denoising were more rigorously examined with permutation tests, which compared the influences of different denoising methods on the uncertainties of the DTI data. Experimental procedures were conducted as follows. Firstly, the DWI data were computed from the denoised and uncorrupted DTI data. Secondly, residuals were obtained by calculating the differences between the DWIs from the denoised DTI data and those from the uncorrupted DTI data. The residuals were then randomly permuted and added to the uncorrupted DWI data. This process was repeated 1,000 times to produce 1,000 new DWI datasets, from which DTI data were derived. The 1,000 samples of each of the six independent tensor components were fitted with normal distributions, and the mean and 95% confidence intervals for each component were calculated. Finally, an ellipsoid was constructed corresponding to the mean DTI data, which was sandwiched by ellipsoids corresponding to the upper and lower bounds of the confidence interval.

Parameters of the proposed denoising were set as follows. In NLM, the parameter *h* controls the decay of the exponential function, which impacts the degree of filtering. Typically, the value of *h* is selected on the basis of the variance of noise (14). In the present study, it was manually tuned to 30, since this value had produced optimal results in preliminary trials. The neighborhood was empirically defined to be a 5×5 window [the same window size was used for Gaussian filtering (GF)], which was also demonstrated to yield optimal results. The parameters of UNLM in DWI space were implemented using the optimal ones suggested by the author (19).

### Experiments with in vivo human DTI data

To assess the performance of the proposed algorithm on *in vivo* data, DWI data were acquired from a healthy human volunteer using a 3T Philips Intera Achieva MR scanner (Philips Medical Systems, Best, Netherlands) and an eight-element SENSE coil. A single shot, echo-planar pulsed gradient spin-echo imaging sequence was used, and diffusion weighting was performed along 32 non-collinear directions with a *b* value of 1,000 sec/mm^2^. A total of 64 contiguous, 2-mm-thick slices with a matrix size of 128×128 were acquired from a field of view of 256×256 mm^2^, yielding an in-plane pixel size of 2×2 mm^2^. Three repeated scans were obtained from each subject, which were motion and distortion corrected and then averaged using the Philips diffusion registration PRIDE tool (Release 0.4; Philips Medical Systems). Diffusion tensors were estimated from the averaged DWI data using a linear least-square fitting procedure (1), from which FA maps were computed. Prior to the study, the subject provided informed consent for the study protocol that had been approved by the local ethics committee of West China Hospital.

The same four methods were utilized with the *in vivo* dataset and the parameter settings were the same as for the synthetic experiments. Denoising results were evaluated visually and quantitatively, on the basis of the tensor geometry, the mean angular deviation of PD and the mean deviation of FA, as before.

## Results

### Results with simulated DTI data

Results of the proposed DTI-NLM method are demonstrated in Fig. 1D–H, with smoothing with GF, weighting with RD, LED and ED, and the UNLM method in DWI space, respectively. In reference to the corrupted image in Fig. 1C, the results indicated that all the denoising methods restored the tensor shape and orientation, although to varying degrees. Compared with the traditionally used GF (Fig. 1D), which smoothed DTI data in homogeneous regions and blurred edges simultaneously, the proposed NLM method effectively preserved the edges while greatly reducing noises throughout the image. This was manifested in the consistency of the shape and orientation with the original noise-free data, even at the edge of the fiber bundle, as opposed to the spreading of these shapes into the background regions by GF.

Among the different weighting schemes using NLM, the RD and LED methods (Fig. 1E and F) yielded the best results due to the use of full information from the tensor. By contrast, ED weighting produced the poorest results due to the reliance on only partial tensor information in computing the weight. In particular, the UNLM in DWI space achieved reasonable results which restored shape and orientation simultaneously.

Quantitative evaluations of the smoothing effects are shown in Table I, which lists the mean deviations of PD and FA for each of the methods implemented. The results demonstrated that the effects of denoising with RD and LED weighting were similar, as expected, and were better than the effects achieved with ED. The mean deviations of PD for the RD and LED methods decreased by more than one degree when compared with the noised DTI data. By contrast, the result from UNLM in DWI space was also comparative and achieved a similar result to RD and LED. In addition, GF yielded the largest improvement in the deviation of FA, which was not unexpected since smoothing of a scalar field can be reasonably achieved through neighborhood averaging.

**Figure 2:**
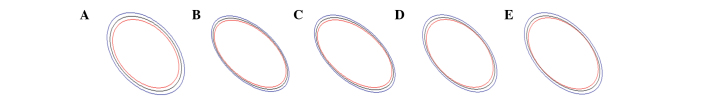
Tensor uncertainties prior to and following various denoising methods. The mean value is denoted in black and the 95% confidence interval is denoted in red and blue. Tensor uncertainty after (A) 5% Rician noise corruption, and denoising with (B) RD, (C) LED and (D) ED weighting and (E) UNLM in DWI space. RD, Riemannian distance; LED, Log-Euclidean distance; ED, Euclidean distance; UNLM, unbiased non-local mean; DWI, diffusion-weighted image.

Changes in the tensor uncertainties following denoising are shown in Fig. 2. The voxel selected was the one with the largest FA, as shown in Fig. 1. Fig. 2A demonstrates the upper (blue) and lower (red) bounds of the tensor following corruption with 5% Rician noise. Following denoising, the tensor uncertainty exhibited varying levels of changes. Among all the denoising methods, RD and LED weighting yielded the tensor with the smallest confidence intervals, attesting again to their better denoising performance in comparison with the other methods.

### Results with in vivo human DTI data

**Figure 3:**
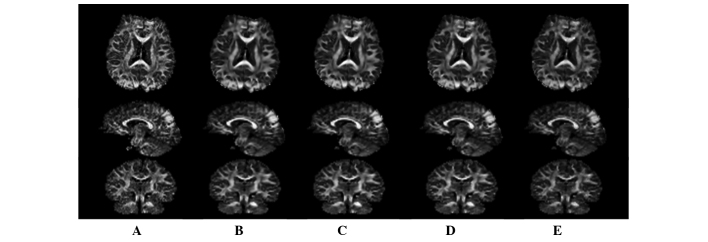
*In vivo* FA maps prior to and following denoising. FA map of (A) original image, and denoising with (B) RD, (C) LED and (D) ED weighting and (E) UNLM in DWI space. Upper row, axial plane; middle row, sagittal plane; lower row, coronal plane; FA, fractional anisotrophy; RD, Riemannian distance; LED, Log-Euclidean distance; ED, Euclidean distance; UNLM, unbiased non-local mean; DWI, diffusion-weighted image.

**Figure 4:**
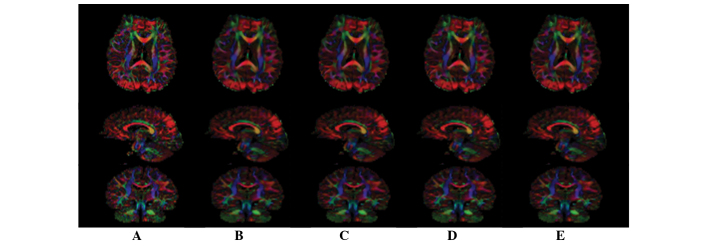
*In vivo* PD maps prior to and following denoising. PD map of (A) original image, and denoising with (B) RD, (C) LED and (D) ED weighting and (E) UNLM in DWI space. Upper row, axial plane; middle row, sagittal plane; lower row, coronal plane; PD, principal direction; RD, Riemannian distance; LED, Log-Euclidean distance; ED, Euclidean distance; UNLM, unbiased non-local mean; DWI, diffusion-weighted image.

Figs. 3 and 4 demonstrate the effects on the shape and orientation information, respectively, using the proposed DTI-NLM method with the three weighting strategies and UNLM DWI denoising in the human brain. Figs. 3A and 4A show the FA and PD maps, respectively, of one slice of the *in vivo* human DTI dataset, which demonstrated marked artifacts resulting from noise. Figs. 3B–E and 4A–E show the FA and PD maps, respectively, following denoising using the four aforementioned methods. Among the NLM-DTI based denoising methods, the RD and LED weighting methods achieved visually the most appealing effects in the FA and PD maps, which may be attributable to their use of full tensor information in similarity weighting. The ED weighting method also restored the FA and PD maps well, with a little residual noise artifacts observed, but somewhat clearer structure boundaries.

**Figure 5:**
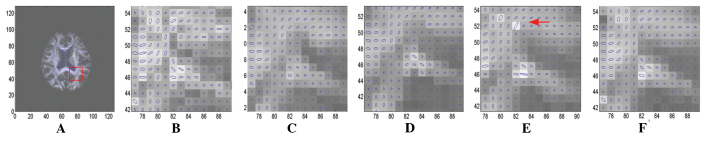
Visual assessment of the denoising effects on *in vivo* DTI data. The slice shown is the same as in Fig. 3 and 4, and each tensor is represented by an ellipsoid. (A) A slice of the *in vivo* dataset. (B) Enlarged view of the boxed region in (A). Denoising with (C) RD, (D) LED and (E) ED weighting and (F) UNLM in DWI space. DTI, diffusion tensor imaging; RD, Riemannian distance; LED, Log-Euclidean distance; ED, Euclidean distance; UNLM, unbiased non-local mean; DWI, diffusion-weighted image.

To assess the denoising effects in the tensor structure, the same section presented in Fig. 3 was selected. Fig. 5 shows the tensor ellipsoids of the restoration result using the four aforementioned denoising methods. In Fig. 5, the background is the FA map of the slice and the tensor is represented by a 3D ellipsoid projected onto the FA plane. Fig. 5B–F show an enlarged view of the boxed region in Fig. 5A. The results indicate that with the original noised image, the ellipsoids were disarrayed and the tensors appeared to have marked irregularity in shape and direction. Following the use of the proposed NLM methods with the RD and LED weighting strategies (Fig. 5C and D), the structures were well restored, and the structure and orientation were regularly arranged, with the exception of a few artifacts. In addition, the NLM-DTI method using ED weighting accomplished reasonable results, however, an evident error tensor was observed (red arrow in Fig. 5E). This may have been caused by the inaccuracy of the tensor weighting from the partial comparison using ED. It should be noted that the UNLM in DWI method also yielded a significant improvement in the tensor shape and orientation. Overall, the effects of these methods on the *in vivo* data were similar to those on the simulated data.

### Computational efficiency

The computational efficiency of the proposed NLM-DTI algorithm with the three different weighting schemes and UNLM in DWI space is demonstrated in Table II. The time consumption primarily depended on the size of the dataset. In addition, DWI denoising required duplicating the procedure for all the directions, which increased the computational time. Under the parameter settings used in the present study and on a notebook computer with an Intel Core (TM) i7 CPU, 4GB RAM, scripted in MATLAB, the running time of the proposed method was <30 sec, which should satisfy the majority of research and clinical applications.

## Discussion

In the present study, a novel DTI denoising method in the tensor space is proposed. Following the widely recognized NLM method, DTI data were denoised by averaging the tensors in a specific neighborhood according to their similarity to the tensor in the center. Under the proposed NLM framework, three weighting schemes corresponding to different features extracted from the tensor, as well as GF in DTI space and UNLM denoising in DWI space, were compared quantitatively and qualitatively with synthetic and real human brain DTI datasets.

The results of these experiments demonstrated that the proposed method is able to suppress noise and preserve explicit boundaries in DTI data, with the extent varying depending on the similarity weighting scheme used. Specifically, the denoising performance relied primarily on the accuracy of tensor comparison. When the tensor was compared on the basis of a greater number of structural features, as implemented in RD and LED, the proposed NLM method achieved the best effect. By contrast, when the comparison used fewer structural features, as implemented in ED, the denoising effect was markedly decreased.

As previously discussed, DTI denoising may be broadly divided into two categories: Denoising in the DWI space and denoising in the DTI space. Compared with DWI denoising, DTI denoising is much more convenient to implement and is able to achieve similar denoising results for orientation and structure information. Unlike DTI denoising, which involves only six independent parameters, DWI data normally entail a much greater number of directions and the denoising procedure has to be duplicated for all the directions. As the application of HARDI increases (13), the computational burden is likely to become the critical disadvantage of DWI denoising due to the hundreds of scanning directions and different *b* values. In addition, due to the nonlinearity between the DWI and DTI spaces, optimal denoising results in the DWI space are not necessarily optimal in the DTI space. Therefore, denoising DTI directly is able to achieve the most intuitive restoration results and convenience for subsequent applications.

Finally, it should be considered that the proposed method does not come without limitations, despite its great potential. Although denoising in DTI space is timesaving when compared with DWI denoising, the NLM method itself is generally computationally intensive. This can be resolved by improving the NLM framework (23) or by implementing the smoothing process with more advanced computational techniques. In conclusion, the proposed method smooths DTI data in the tensor space with an NLM method, which extracts information from the tensor for computing the weight for averaging. The efficacy and advantages of the technique have been demonstrated by experiments with synthetic and *in vivo* human DTI data. With the application of an affine invariant matrix, which encapsulates the comprehensive information of the diffusion tensor, NLM-DTI denoising is able to achieve comparative restoration results to resemble the denoising method in DWI space. In addition, the time efficiency demonstrates the practical use for this method as a routine denoising algorithm.

## Figures and Tables

**Table I:** Mean deviations of PD and FA following the addition of 5% Rician noise and the denoising of synthetic data.

Parameter	Add 5% noise	LED	RD	ED	UNLM in DWI	GF
Mean deviation of PD	5.2317	3.9814	4.1129	4.4398	4.1349	4.4912
Mean deviation of FA	0.0573	0.0487	0.0496	0.0512	0.489	0.0459

PD, principal direction; FA, fractional anisotropy; RD, Riemannian distance; LED, Log-Euclidean distance; ED, Euclidean distance; UNLM, unbiased non-local mean; DWI, diffusion-weighted image; GF, Gaussian filtering.

**Table II:** Running time of synthetic and *in vivo* data (sec).

Data	RD	LED	ED	UNLM in DWI
Synthetic	3	3	3	61
*In vivo*	23	24	23	468

RD, Riemannian distance; LED, Log-Euclidean distance; ED, Euclidean distance; UNLM, unbiased non-local mean; DWI, diffusion-weighted image.
